# Identification of a Small Secretoneurin Derivative That Inhibits CaMKIIδ Activity

**DOI:** 10.1111/jcmm.70900

**Published:** 2025-10-22

**Authors:** Ilde Rugolo, Xin Shen, Thea Parsberg Støle, Anna Bergan‐Dahl, Ornella Manfra, Marianne Lunde, Geir Christensen, Bjørn Dalhus, William E. Louch, Anett H. Ottesen, Helge Røsjø, Cathrine Rein Carlson

**Affiliations:** ^1^ Akershus Clinical Research Center (ACR), Division of Research and Innovation Akershus University Hospital Lørenskog Norway; ^2^ Institute for Experimental Medical Research Oslo University Hospital and University of Oslo Oslo Norway; ^3^ K.G. Jebsen Center for Cardiac Biomarkers, Institute of Clinical Medicine, Faculty of Medicine University of Oslo Oslo Norway; ^4^ Department of Microbiology Oslo University Hospital Oslo Norway; ^5^ Department of Medical Biochemistry, Institute for Clinical Medicine University of Oslo Oslo Norway

**Keywords:** arrhythmias, CaM, CaMKII, heart failure, phospholamban, RYR, secretoneurin, SN

## Abstract

Ventricular arrhythmias, a major cause of sudden cardiac death, are driven by Ca^2+^ imbalance in cardiac myocytes, often linked to the overactivation of CaMKIIδ (Ca^2+^/calmodulin‐dependent protein kinase II delta). As such, inhibiting CaMKIIδ represents a promising therapeutic strategy. Based on our previous finding that native secretoneurin (SN) is a weak CaMKIIδ inhibitor, we aimed to develop a more potent derivative of SN to effectively counter aberrant Ca^2+^ handling and arrhythmia risk. Various regions of SN were tested for CaMKII binding, identifying the core region as the sequence with the strongest binding capacity. This region was subsequently optimised with two phenylalanine substitutions, resulting in the SN derivative SN‐db‐short. Structural homology modeling and ELISA‐based assays revealed that SN‐db‐short bound both the substrate‐binding (S‐site) region of CaMKIIδ, in addition to the ATP‐binding region, with 8‐fold stronger binding compared to SN. Surface plasmon resonance experiments confirmed that SN‐db‐short exhibited a higher association rate and affinity for CaMKIIδ compared to SN. Consistent with only a partial calmodulin binding motif, SN‐db‐short showed no calmodulin binding, indicating selective CaMKIIδ inhibition. In functional studies, SN‐db‐short inhibited CaMKIIδ‐mediated phosphorylation of ryanodine receptor 2 and appeared more effective than SN in reducing the incidence of Ca^2+^ sparks and Ca^2+^ waves. SN‐db‐short also more markedly inhibited CaMKIIδ phosphorylation of phospholamban, slowed Ca^2+^ reuptake, and reduced the magnitude of Ca^2+^ transients during isoproterenol stimulation. SN‐db‐short effectively inhibits CaMKIIδ and significantly counters aberrant Ca^2+^ handling in cardiomyocytes. Thus, this optimised peptide holds therapeutic potential for reducing the risk of ventricular arrhythmias.

## 1 Introduction

Calcium (Ca^2+^)‐calmodulin‐dependent kinase II (CaMKII) is a serine/threonine kinase crucial for regulating Ca^2+^ signalling in various cell types, including cardiomyocytes. Among its isoforms, CaMKII delta (CaMKIIδ) is the predominant form expressed in the heart, where it plays an essential role in excitation‐contraction coupling (ECC) by modulating calcium cycling [1, 2]. Specifically, CaMKIIδ phosphorylates key Ca^2+^‐handling proteins, including the ryanodine receptor (RYR) and phospholamban (PLN), thereby regulating the release and reuptake of Ca^2+^ during the cardiac cycle [3].

While CaMKIIδ is critical for normal cardiac function, its dysregulation has been associated with cardiac pathologies, including hypertrophy, arrhythmias, and heart failure [4, 5, 6]. Animal studies have shown that selective inhibition of CaMKIIδ improves cardiac function, reduces arrhythmias, and prevents adverse remodelling. These studies provide strong evidence for the therapeutic potential of CaMKIIδ inhibitors [5, 7, 8]. However, current CaMKII inhibitors are limited by off‐target effects, poor bioavailability, and inefficient internalisation into cardiomyocytes, reducing their translational viability [6, 9].

We have previously shown that the peptide secretoneurin (SN) is an endogenous inhibitor of CaMKIIδ that protects cardiomyocytes against Ca^2+^ mishandling and attenuates Ca^2+^‐dependent arrhythmias [10, 11]. SN is a small neuropeptide (33 aa) belonging to the granin protein family and produced from secretogranin II (SgII) by endoproteolytic cleavage [12]. Since circulating SN levels are elevated in patients with heart failure and correlate with a poor outcome, SN is suggested to be produced as a compensatory mechanism [10]. However, physiological concentrations of SN demonstrate relatively weak inhibitory effects on CaMKIIδ activity [10].

Accordingly, we aimed to identify a derivative of SN with enhanced binding affinity and inhibitory potency for CaMKIIδ. We hypothesised that an optimised version of SN would more effectively counter aberrant calcium handling underlying arrhythmogenesis.

## 2 Materials and Methods

### 2.1 Peptide Synthesis

Native SN and a variety of SN derivative peptides were synthesised with 80%–98% purity by Genscript Corp (Piscataway, NJ, USA) with the following sequences:

- SN: TNEIVEEQYTPQSLATLESVFQELGKLTGPNNQ (SgII 154–186 [SgII 182–214 with signal peptide, UniProt P13521])
- SN (1–20): TNEIVEEQYTPQSLATLESV
- SN (7–26): EQYTPQSLATLESVFQELGK
- SN (14–33): LATLESVFQELGKLTGPNNQ
- SN‐db‐short [SN (7–26, L17F, V20F)]: EQYTPQSLAT **F** ES **F** FQELGK
- Scram: QFQSEKLFSGEPFQTYLETA (control for SN‐db‐short)
- SN (7–26, L14F, L17F, V20F): EQYTPQS **F** AT **F** ES **F** FQELGK
- SN (7–26, S13D, L17F, V20F): EQYTPQ **D** LAT **F** ES **F** FQELGK

Other synthesised peptides included:

- PLN (1–30): MEKVQYLTRSAIRRASTIEMPQQARQNLQN
- RYR (2798–2827): MALYNRTRRISQTSQVSVDAAHGYSPRAID
- CN21: KRPPKLGQIGRSKRVVIEDDR
- CN27: KRPPKLGQIGRAKRVVIEDDRIDDVLK
- AKAP18δ‐N1: PQGNVPQGNPKRSKENRGDR
- AIP: RKKALRRQGAVDAL

The peptides were synthesised with or without an N‐terminal biotin‐ahx or FITC (Fluorescein‐5‐isothiocyanate)‐ahx (Genscript).

### 2.2 Recombinant Proteins and Fragments

Recombinant human His‐CaMKIIδ‐T287D and rat His‐CaMKIIδ‐T287D full‐length proteins (T287D mutated to mimic active kinases) and rat His‐CaMKIIδ (1–74), rat His‐CaMKIIδ (1–165), rat His‐CaMKIIδ (1–282), rat His‐CaMKIIδ‐T287D (1–311), and rat His‐CaMKIIδ (69–282) recombinant fragments were generated by Genscript Corp.

### 2.3 ELISA

A 96‐well microplate was coated with 1 μg/well of rat or human recombinant His‐CaMKIIδ‐T287D or CaMKIIδ fragments (custom made by Genscript) in 1× PBS overnight at 4°C with gentle tilting. Wells were washed with 1× PBS‐t (0.1% Tween‐20) and blocked with 0.5% gelatin (G‐1890 Gelatin type A, Sigma Merck, Darmstadt, Germany) or BSA (805095 30% BSA (Rinderalbumin 30%), Norsk Labex, Høvik, Norway) for 1 h at room temperature (RT), then incubated with 5 μL of biotinylated peptide (1 mM) for 2 h at 37°C with gentle tilting. The wells were then washed five times with 1× PBS‐t. To detect binding, the wells were incubated with a monoclonal anti‐biotin‐HRP conjugated antibody (1:5000 dilution, A‐0185, Sigma) for 30 min at RT. The washing steps were repeated, and the plate was incubated with the Ultra TMB solution (1‐step Ultra TMB‐ELISA Substrate Solution 34028, Thermo Fischer Scientific, Waltham, MA, USA) for 30 min at RT with gentle tilting. The reaction was stopped by adding 2 N HCl (100 μL), and the signal was measured at 450 nm by a plate reader (Hidex Sense Microplate Reader, Åbo, Finland). For the competition assay, 20 μL (1 mM) of T‐site binding peptides AKAP18δ‐N1, CN21 or CN27 (without any biotin tags) were incubated in the same well with 5 μL (1 mM) of SN biotinylated peptides.

### 2.4 Sequence Alignment

**FIGURE 1:**
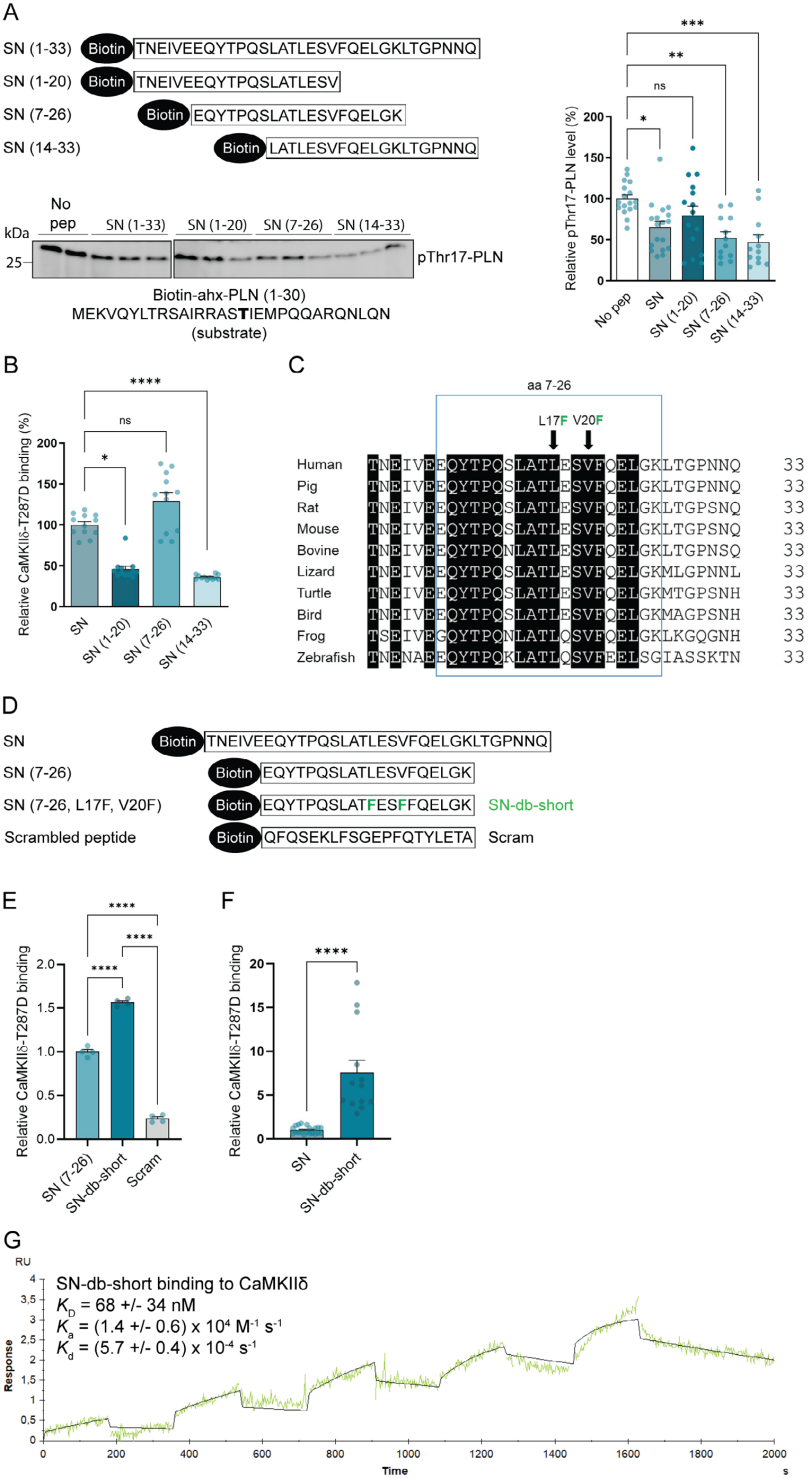
Identification of a small SN derivative that inhibits and binds CaMKIIδ stronger than native SN. (A) Upper panel: Schematic illustration of biotinylated SN (1–33) and SN derived peptides, covering amino acids 1–20, 7–26 and 14–33, used in the CaMKIIδ activity assay. Lower panel: CaMKIIδ phosphorylation of biotin‐ahx‐PLN (1–30) analysed with and without the presence of SN or the different SN derived peptides. Bar charts present mean values + SEM. Normal distribution was confirmed by Kolmogorov–Smirnov test. Significant differences were examined by ordinary one‐way ANOVA with Tukey's multiple comparisons test (*n* = 12–18). *p < 0.05, **p < 0.01, ***p < 0.001, ns, not significant. (B) Binding of the different biotin‐SN derived peptides to His‐CaMKIIδ‐T287D (coated in wells) analysed by an ELISA‐based method. Bar charts present mean values + SEM. Significant differences were examined by Kruskal–Wallis test with Dunn's multiple comparisons test (*n* = 12). *p < 0.05, ****p < 0.0001, ns, not significant. (C) Alignment of secretogranin II (SgII) sequences for different species corresponding to human SN (1–33) using Align (Uniprot). The box indicates the most conserved region across the species (identical amino acids are in black). L17 and V20 were mutated with phenylalanines (F) to obtain SN‐db‐short (indicated by arrows). (D) Schematic presentation of biotinylated SN, SN (7–26), SN (7–26, L17F, V20F) (SN‐db‐short) and a scrambled negative control peptide (Scram) used in E and F. (E) Binding of biotinylated SN‐db‐short and SN (7–26) to His‐CaMKIIδ‐T287D (coated in wells) analysed by an ELISA‐based method. Scram was used as negative control. Bar charts present mean values + SEM. Normality of distribution was confirmed by Shapiro–Wilk test. Significant differences were examined by ordinary one‐way ANOVA with Tukey's multiple comparisons test (*n* = 4). ****p < 0.0001. (F) Binding of biotinylated SN and SN‐db‐short to His‐CaMKIIδ‐T287D (coated in wells) analysed by an ELISA‐based method. Bar charts present mean values + SEM. Normality of distribution was confirmed by D'Agostino and Pearson test. Significant differences were examined by unpaired *t*‐test (*n* = 13–20). ****p < 0.0001. (G) Surface plasmon resonance (SPR) analysis of biotinylated SN‐db‐short immobilised on a streptavidin chip and recombinant His‐CaMKIIδ‐T287D injected over the chip. The green curve shows the experimental data, and the black curve shows the fit for a 1:1 binding model. The given dissociation equilibrium and rate constants are an average of three experiments.

We used the program Align by Uniprot for alignments. Sequences used for the alignment in Figure 1C were *Homo sapiens* (P13521), *Sus scrofa* (Q5FZP5), *Rattus norvegicus* (P10362), *Mus musculus* (Q03517), *Bos taurus* (P20616), *Varanus merrem* (UPI001CF78B26), *Mauremys reevesii* (UPI00193EFE6E), *Gallus gallus* (A0A1D5NUV0), *Xenopus laevis* (A0A1L8GB41), and *Danio rerio* (A0A8M3AHQ7). Rat CaMKIIδ (P15791) and human CaMKIIδ (Q13557) were used for the alignment in Figure S2C.

### 2.5 Surface Plasmon Resonance

Surface plasmon resonance (SPR) was performed using Biacore X100 at room temperature (Biacore Inc., Uppsala, Sweden). A streptavidin (SA) chip (BR100032, Cytiva, Marlborough, MA, USA) was conditioned with 1× Biacore running buffer (BR100826, Cytiva) in three consecutive 1‐min injections. The 10× buffer contains 0.1 M HEPES, 1.5 M NaCl, 0.03 M EDTA, and 0.5% (v/v) Surfactant P20. Biotin‐ahx‐SN‐db‐short was immobilised at 58 resonance units (RU). Recombinant His‐tagged CaMKIIδ‐T287D was dialysed into running buffer with the addition of 2.5 mM Ca^2+^, as our previous work has indicated that the CaMKIIδ‐SN interaction is Ca^2+^ dependent [11]. A serial dilution was performed, with halving of the concentration of recombinant protein at each step, to produce a range of test concentrations (1, 0.5, 0.25, 0.125 and 0.06 μM). These were injected over the sensor chip surface in single‐cycle mode. The flow rate was set to 30 μL/min for 180 s and the dissociation time was set to 600 s. Sensorgrams were analysed using the Biacore X100 evaluation software with the Langmuir model (assumes one‐to‐one binding).

### 2.6 CaMKII Activity Assay

A CaMKII activity assay was performed using PLN threonine 17 (Thr17‐PLN) or RYR serine 2814 (Ser2814‐RYR) as a substrate (biotin‐ahx‐PLN [1–30], biotin‐ahx‐RYR [2798–2827]). Recombinant human His‐CaMKIIδ‐T287D (Genscript) and biotin‐ahx‐PLN (1–30) or RYR (2798–2827) were incubated with 10 μL of 1 mM biotin‐SN, biotin SN‐db‐short or the scrambled control peptide biotin‐scram in CaMKII kinase buffer (50 mM Hepes, pH 7.5, 150 mM NaCl, 15 mM MgCl_2_, 1 mM CaCl_2_, 0.1% Triton X‐100, 1% BSA, 0.1 mM ATP, 10 μg/mL CaM) for 30 min at 37°C. The reactions were stopped by boiling samples in SDS loading buffer for 5 min at 95°C. The level of CaMKIIδ‐mediated phosphorylation of Thr17‐PLN and Ser2814‐RYR was analysed by immunoblotting.

### 2.7 Isolation of Adult Rat Cardiomyocytes From Left Ventricle

Male Wistar rats (Janvier, France) weighing ~300 g were sedated in an anaesthesia chamber by inhalation of 5% isoflurane and 95% O_2_. The heart was excised under deep surgical anaesthesia, cannulated, and then retrogradely perfused through the aorta on a modified Langendorff setup with cell isolation solution containing (in mM, pH adjusted to 7.4, 37°C): NaCl 130, Hepes 25, D‐glucose 22, KCl 5.4, MgCl_2_ 0.5, NaH_2_PO_4_ 0.4, and a flow rate of 4 mL/min. After the heart was cleaned of blood, collagenase type II (2 mg/mL, Worthington Biochemical Corp, Lakewood, NJ, USA) was added to the perfusate, and the hearts digested for ~10 min. Following digestion, hearts were cut down, and the left ventricle dissected into pieces, which were transferred into 10 mL cell isolation solution with 250 μL BSA (40 mg/mL) and ~0.2 mg Deoxyribonuclease I (Worthington Biochemical Corporation, Lakewood, NJ, USA). After light agitation (~2 min), the supernatant was filtered (200 μm nylon mesh), and isolated cardiomyocytes were allowed to sediment by gravity. The cardiomyocytes were then washed in isolation buffer containing 1 mg/mL BSA, with a stepwise increase in Ca^2+^ concentration up to 0.2 mM. Animal experiments were approved by the Norwegian Research Authority (FOTS ID: 30114).

### 2.8 Pull Down Experiments

In the CaMKII pull‐down assay, the biotinylated peptides (20 μL) were coupled to 50 μL of streptavidin magnetic beads (88816, Thermo Fisher) in PBS for 2 h at 4°C with rotation. The bead‐bound peptides were thereafter incubated with 1 μg of human His‐CaMKIIδ‐T287D in 300 μL of PBS or PBS with 1% BSA for 2 h at 4°C with rotation. The protein complexes attached to the beads were then washed three times in 300 μL of IP buffer (20 mM Hepes, pH 7.5, 150 mM NaCl, 1 mM EDTA, 1% Triton X‐100, supplemented with complete protease inhibitor cocktail and phosphatase inhibitor cocktail) or PBS. Samples were boiled in SDS loading buffer at 95°C for 5 min before western blot analysis.

To test for calmodulin (CaM) binding, recombinant CaM was pulled down after incubation with SN‐db‐short or a peptide representing the CaM binding site in CaMKII (KFNARRKLKGAILTTMLATR, positive control). SN‐db‐short and the CaM‐binding peptide were synthesised in triplicates onto a cellulose membrane by a Multipep automated peptide synthesiser (CEM Corporation, Matthews, NC, USA). The specific spots of the membrane corresponding to SN‐db‐short or the positive control peptide were cut and placed into separate tubes. The membrane pieces were first rehydrated in 100% methanol and subsequently incubated with 1 μg of recombinant CaM (C4874, Sigma Merck) in CaMKII binding buffer supplemented with CaCl_2_ (50 mM Hepes, 150 mM NaCl, 15 mM MgCl_2_, 1 mM CaCl_2_, 0.1% Triton X‐100, 1% BSA) overnight at 4°C with rotation. The supernatants were then removed, and membrane pieces were washed three times in CaMKII binding buffer. Finally, they were boiled in 2× SB buffer at 95°C for 5 min before western blot analysis.

### 2.9 Western Blot Analysis

Lysates and immunoprecipitates were separated on 12% or 4%–15% Criterion TGX precast gels (5671044 and 5671084, Biorad, Hercules, CA, USA), blotted onto PVDF membranes (1704157 by Biorad or 03010040001 by Sigma Merck) and reversibly stained with Revert 700 Total Protein Stain (Li‐COR Biosciences, Lincoln, NE, USA). Next, the membranes were blocked in 5% non‐fat dry milk (A0830, VWR, Radnor, PA, USA) or 1× casein (11921673001 Western Blocking Reagent, Sigma Merck) in TBS‐t (0.2 M Tris‐base, 1.5 M NaCl, 40 mM HCl, pH 7.5 supplemented with 0.1% Tween‐20) for 1 h at RT with gentle tilting. Thereafter, the membranes were incubated overnight with primary antibodies at 4°C with gentle tilting. Following three washing steps of 10 min in TBS‐t, membranes were incubated with HRP‐conjugated secondary antibody for 1 h at RT. Blots were developed using ECL (RPN2236, GE Healthcare, Chicago, IL, USA) after four washing steps of 10 min in TBS‐t and the chemiluminescence signals were detected by Azure Biosystems 600 (Dublin, CA, USA). Membranes were re‐probed after stripping in Restore Western Blot Stripping Buffer (PIER21603, Thermo Fisher Scientific) for 5 or 10 min and three washing steps of 5 min. ImageJ [13] was used to quantify the blots.

### 2.10 Antibodies Used for Immunoblotting and Immunoprecipitation

Immunoblotting and immunoprecipitations were conducted using anti‐pThr17‐PLN (1:5000 dilution, A010‐13, Badrilla, Leeds, UK), anti‐PLN (1:3000 dilution, MA3‐922, Thermo Fisher Scientific), anti‐pSer2814‐RYR (1:5000 dilution, A010‐31, Badrilla), anti‐RYR (1:1000 dilution, MA3‐916, Thermo Fisher Scientific), anti‐CaMKIIδ (1:1000 dilution, custom‐made from GenScript) and anti‐SN‐db‐short (1:1000 dilution, custom‐made from GenScript). Horseradish peroxidase‐(HRP) conjugated anti‐mouse (1:3000 dilution, NA931V, GE Healthcare), anti‐rabbit (1:3000 dilution, NA934V, GE Healthcare) and monoclonal anti‐biotin (1:5000 dilution, A‐0185, Sigma Merck) were used as secondary antibodies.

### 2.11 Peptide Internalisation in Adult Rat Cardiomyocytes

Adult cardiomyocytes isolated from rat left ventricle were incubated with FITC‐labelled SN‐db‐short or FITC‐labelled SN (15 μM) in 0.2 mM Ca^2+^ cell isolation solution for 2 h at RT. After two washing steps in PBS and a 10 min incubation with PBS containing DAPI readymade solution (1:200 dilution, MBD0015, Sigma Merck), cells were analysed using an LSM 800 confocal microscope. FITC was excited with a 488 nm laser, and fluorescence emission was detected between 500 and 550 nm. DAPI fluorescence was excited at 405 nm, and emission was detected between 450 and 550 nm. Images were processed uniformly in ImageJ by multiplying the signal intensity by a constant factor across all samples. Channels were then merged to generate the final composite images.

Whole‐cell fluorescence quantification was performed using ImageJ. FITC acquisition images from three independent cardiomyocyte isolations, acquired under identical conditions, were analysed. To improve visualisation, all images were multiplied by the same intensity factor. The ROI (region of interest) of each cell was manually delineated using the polygon selection tool, and the mean fluorescence intensity (defined as the sum of pixel values divided by the number of pixels within the ROI) was measured and plotted.

### 2.12 Ca^2+^ Imaging and Analysis

Adult rat cardiomyocytes were treated with 10 μM of SN, SN‐db‐short or scram in 0.2 mM Ca^2+^ cell isolation solution for 1 h. These loading concentrations are higher than those used in our previous studies of SN, where we had sought to examine endogenous, circulating levels [10, 11], rather than potentially therapeutic levels applied exogenously. For the final 15 min of the loading period, the incubation solution was supplemented with fluo‐4 AM (20 μmol/L) with or without isoproterenol (ISO, 100 nM). Experiments were then started, where the cardiomyocytes were perfused with Hepes‐Tyrode (HT) solution (140 mM NaCl, 0.5 mM MgCl_2_, 5.0 mM HEPES, 5.5 mM glucose, 0.4 mM NaH_2_PO_4_, 5.4 mM KCl and 1.8 mM CaCl_2_, pH 7.4, 37°C). Cells were field stimulated via a pair of platinum electrodes paced at 1 Hz, using 3 ms supra‐threshold current pulses. Ca^2+^ transients were recorded in linescan mode using a Zeiss LSM 7 Live confocal microscope (Jena, Germany), at a frame rate of 1.5 ms and a pixel size of 0.16 μm. These recordings were normalised to resting fluorescence values to create F/F_0_ images and assess Ca^2+^ transient amplitude [14]. The rate of Ca^2+^ transient decline was assessed by measurements of the decay time constant (Tau). Spontaneous sparks and waves were measured during a 9 s pause in the electrical excitation. Ca^2+^ spark analysis was performed using IOCBIO Sparks [15].

To estimate SR Ca^2+^ content, Ca^2+^ release was triggered by rapid exposure to 10 mM caffeine (Sigma) and recorded by whole‐cell photometry (Photon Technology International, Monmouth Junction, NJ, USA) with a 40× oil objective. Cells were first stimulated at 1 Hz in Hepes‐Tyrode (HT, see the paragraph above) solution containing CaCl_2_ until at least three stable Ca^2+^ transients were recorded. The superfusion was then rapidly switched to an HT solution containing caffeine, while electrical stimulation was simultaneously stopped. Caffeine application was maintained for a few seconds and discontinued once the Ca^2+^ signal returned to a steady state. The magnitude of the caffeine transient was used to estimate SR Ca^2+^ content, while the rate constant for the decline of the caffeine transient (i.e., *λ*_CAF_ = 1/*τ*_CAF_) was used to estimate extrusion of Ca^2+^ by the Na^+^‐Ca^2+^ exchanger and plasma membrane Ca^2+^ ATPase [16]. The tau values used in these measurements were obtained using single‐exponential fits and Clampfit software (version 10.4).

### 2.13 Structural Model

The structural models of peptides binding to CaMKIIδ were based on the previous model of SN binding to the S‐site [10] and analysed and visualised by PyMol (Schrodinger LLC).

### 2.14 Statistics

All datasets with a small n (*n* < 8) were tested for normality of distribution using Kolmogorov–Smirnov, Shapiro–Wilk, or D'Agostino and Pearson normality test (GraphPad Prism 9). Differences between groups with normally distributed data were analysed using ordinary One‐way ANOVA with Dunnett, Holm‐Sidak, or Tukey multiple comparisons test, or unpaired Student's *t* test for simple 2‐group comparison. Non‐normal distributions were examined by Mann–Whitney *U* test, or Kruskal–Wallis with Dunn's multiple comparisons test. *p* < 0.05 was considered statistically significant. Representative immunoblots were selected to represent the means of the quantified data. Representative images were selected by eye, based on favourable signal/noise ratios.

## 3 Results

### 3.1 Identification of a Small SN Derivative That Inhibits and Binds CaMKIIδ Stronger Than SN

First, we performed a kinase activity assay to investigate the region of the native SN sequence that exhibited the strongest CaMKIIδ inhibition (Figure 1A, illustrated in the upper panel). These experiments were executed by employing recombinant CaMKIIδ‐T287D, which mimics the active kinase (the CaMKII activation process is illustrated in Figure S1A–C) [17]. These experiments were performed with or without the presence of three overlapping peptides covering the SN sequence: amino acids 1–20, 7–26, and 14–33. A biotinylated PLN (1–30) peptide was used as a substrate. Immunoblotting with pThr17‐PLN specific antibodies showed that SN (7–26) and SN (14–33) reduced the level of CaMKIIδ‐dependent phosphorylation of Thr17‐PLN to a similar level as that obtained with native SN (Figure 1A, lower panel, and graph at right). The CaMKIIδ‐T287D binding capacity of the three biotinylated SN‐derived peptides (aa 1–20, 7–26, and 14–33) was further analysed using an ELISA‐based assay, where wells were coated with recombinant CaMKIIδ‐T287D. Whereas SN (1–20) and SN (14–33) both bound more weakly to CaMKIIδ‐T287D, SN (7–26) showed a tendency towards stronger binding than native SN (Figure 1B), suggesting that the N‐ and/or C‐terminal regions of native SN may cause steric hindrance for the CaMKII binding site. Interestingly, alignment of SgII sequences from different species showed that aa 7–26 is derived from the most conserved part of SN (Figure 1C).

In an attempt to obtain a peptide with even stronger CaMKIIδ binding capacity, we further introduced two phenylalanine (F) mutations into SN (7–26). These two mutations have previously been shown to increase CaMKIIδ binding to SN [10]. As indicated by an ELISA‐based assay, these two mutations in the novel SN peptide (7–26, L17F, V20F) (Figure 1D) also showed 1.5‐fold CaMKIIδ‐T287D binding capacity compared to SN (7–26) (Figure 1E), and 8‐fold compared to native SN (Figure 1F). The novel peptide sequence was EQYTPQSLAT**F**ES**F**FQELGKL and named SN‐db‐short (Figure 1D). We also demonstrated increased binding of SN‐db‐short to recombinant human CaMKIIδ‐T287D vs. native SN in additional pull down and ELISA experiments (Figure S2A,B, respectively). Consistently, alignment analysis showed that rat and human CaMKIIδ have identical catalytic domains (Figure S2C).

Finally, the affinity and kinetics of the SN‐db‐short‐CaMKIIδ interaction were analysed using surface plasmon resonance (SPR). Biotinylated SN‐db‐short was immobilised on an SA chip and a serial dilution of CaMKIIδ‐T287D was injected over the chip. The interaction was fitted with a 1:1 binding model (Langmuir). The dissociation equilibrium constant (*K*_*D*_) for the SN‐db‐short‐CaMKIIδ interaction was 68 ± 34 nM, with an association rate constant (*k*_a_) of (1.4 ± 0.6) × 10^4^ M^−1^ S^−1^, and a dissociation rate constant (*k*_d_) of (5.7 ± 0.4) × 10^−4^ S^−1^ (Figure 1G). Thus, SN‐db‐short appears to have a higher association rate and affinity for CaMKIIδ compared to previously reported values for native SN: *K*_*D*_ = 8 ± 3 × 10^−8^ M, *k*_a_ = 3 ± 1 × 10^3^ M^−1^ s^−1^, and *k*_d_ = 3 ± 2 × 10^−4^ s^−1^ [10].

### 3.2 SN‐db‐Short Binds to the ATP‐Binding Region and S‐Site in CaMKIIδ Catalytic Domain

**FIGURE 2:**
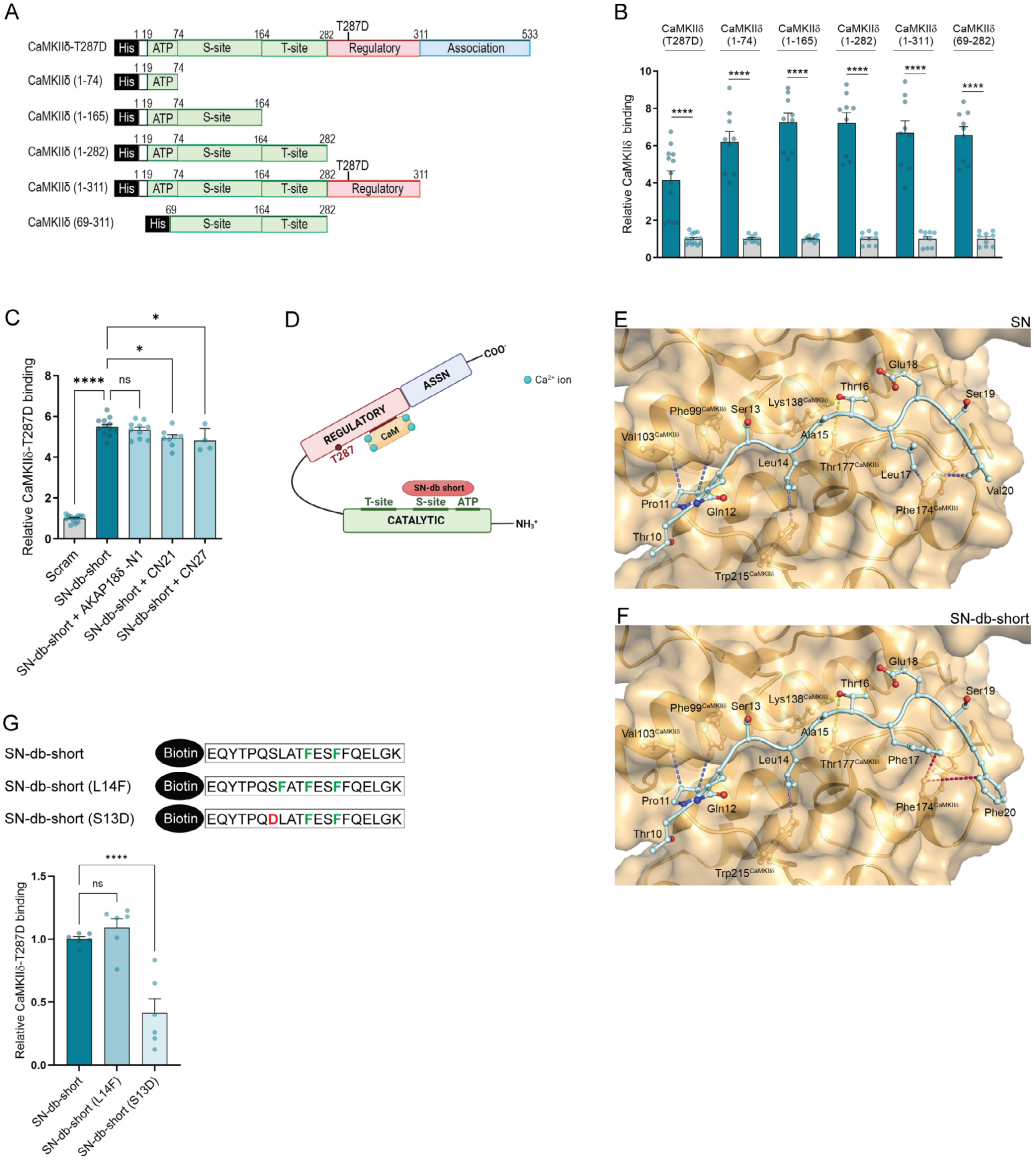
The optimised SN‐db‐short peptide binds to the ATP‐binding site and S‐site in CaMKIIδ. (A) Schematic illustration of recombinant CaMKIIδ‐T287D and various CaMKIIδ fragments used in B. The illustration shows the catalytic domain in green, the regulatory domain in red and the association domain in blue. Notably, the definitions of the S‐ and T‐sites are only loosely defined; S‐site is reported to contain at least Glu97 and Glu140, whereas T‐site contains at least Ile206 and Trp238 [18]. (B) Binding of SN‐db‐short to different CaMKIIδ fragments (coated in wells) analysed by an ELISA‐based method. Values are presented relative to the scrambled control peptide (Scram) (=1). Bar charts present mean values + SEM. Normality of distribution was confirmed by D'Agostino and Pearson test. Significant differences were examined by unpaired *t*‐test (*n* = 9–13). ****p < 0.0001. (C) SN‐db‐short binding to CaMKIIδ analysed by an ELISA‐based competition assay. The T‐site binding peptides AKAP18δ‐N1, CN21, and CN27 were used as competitor peptides. Values are presented relative to the control peptide (Scram). Bar charts present mean values + SEM. Normality of distribution was confirmed by D'Agostino and Pearson test. Significant differences were examined by ordinary one‐way ANOVA with Tukey's multiple comparisons test (*n* = 4–12). *p < 0.05, ****p < 0.0001, ns, not significant. (D) Illustration of SN‐db‐short (red) binding to the S‐site and ATP‐binding region in the catalytic domain (green) of CaMKIIδ. Figure created by BioRender.com. Structural model of the (E) native SN and (F) SN‐db‐short interactions with CaMKIIδ. The core of the peptides is represented in a light blue ball‐and‐stick model, CaMKIIδ is shown in orange. The dashed lines represent the favourable interactions between amino acids (yellow, H‐bond interactions; blue, hydrophobic interactions; pink, aromatic stacking). (G) Upper panel: Schematic illustration of the biotinylated SN‐db‐short, SN‐db‐short (L14F) and SN‐db‐short (S13D) peptides. Lower panel: Binding of the biotinylated peptides to His‐CaMKIIδ‐T287D (coated in wells) analysed by an ELISA‐based method. Bar charts present mean values + SEM. Values are presented relative to SN‐db‐short. Significant differences were examined by Kruskal–Wallis test with Dunn's multiple comparisons test (*n* = 6). **p < 0.01, ns, not significant.

We have previously shown that native SN binds to the S‐site in the catalytic domain of CaMKIIδ [10] (for schematic representations of CaMKII domains and activity states, see Figure S1). To determine which region of CaMKIIδ the SN‐db‐short peptide binds to, we performed an ELISA‐based assay, using recombinant full‐length CaMKIIδ‐T287D protein and different CaMKIIδ‐T287D peptide fragments coated into the wells. The employed fragments corresponded to the ATP‐binding region (aa 1–74) of CaMKIIδ‐T287D, the ATP‐binding region plus the substrate binding site (S‐site) (aa 1–165), the whole catalytic domain (aa 1–282), the catalytic and regulatory domains (aa 1–311), and the S‐ and T‐sites (the threonine 287 binding segment) without the ATP binding region (aa 69–282) (illustrated in Figure 2A). Interestingly, SN‐db‐short binding was detected for all fragments, including the CaMKIIδ (1–74) fragment (Figure 2B), indicating that SN‐db‐short binds to the ATP‐binding region as well as to the S‐site. A biotinylated scrambled control peptide (scram) was used as a negative control.

To investigate whether SN‐db‐short also bound to the T‐site in the catalytic domain of CaMKIIδ, an ELISA‐based competition assay was performed. Recombinant rat CaMKIIδ‐T287D was coated into wells, with the addition of biotin‐SN‐db‐short and different T‐site binding peptides, including A‐kinase anchoring protein 18 delta‐N1 (AKAP18δ‐N1) [19], CN21, and CN27. The latter two peptides are derived from the natural CaMKII inhibitor protein [20] and were applied without the inclusion of biotin tags. As shown in Figure 2C, SN‐db‐short binding to CaMKIIδ‐T287D was only marginally outcompeted by any of the three competitor peptides, suggesting that SN‐db‐short is not a true T‐site binding peptide. Indeed, although the presence of CN21 or CN27 led to a small reduction in CaMKIIδ‐T287D binding to SN‐db‐short, this reduction was minimal compared to our previous competition data with true T‐site binding peptides (75% reduction) [19]. Notably, the CN21 and CN27 peptides have been reported to be long enough to occupy a portion of the S‐site in CaMKIIδ [20], and thus likely also explain the small reduction in the CaMKIIδ binding of SN‐db‐short. Taken together, our data indicate that SN‐db‐short inhibits CaMKIIδ activity through its binding to the ATP‐binding site and S‐site in the CaMKIIδ catalytic domain (illustrated in Figure 2D). Given the proximity of the two sites, it is likely that a single SN‐db‐short molecule can bind to and block both sites—an assumption that is consistent with the 1:1 Langmuir binding model we observed in Biacore experiments (Figure 1G).

Finally, structural modelling of native SN and SN‐db‐short into the peptide‐binding groove of CaMKIIδ (Figure 2E,F, respectively), showed that whereas Leu17^SN^ and Val20^SN^ might form hydrophobic interactions with Phe174^CaMKII^ (Figure 2E), the two mutations Phe17^SN‐db‐short^ and Phe20^SN‐db‐short^ most probably form even more favourable hydrophobic interactions by aromatic stacking with Phe174^CaMKII^ (Figure 2F) [21]. The model predictions were validated by mutating key residues in SN‐db‐short, and subsequent ELISA analysis (Figure 2G). The L14F mutation appears favourable, providing similarly strong affinity for CaMKIIδ as SN‐db‐short, while a repulsive force from CaMKIIδ Glu97 makes the 13D mutation unfavourable.

### 3.3 SN‐db‐Short Does Not Bind to Calmodulin

**FIGURE 3:**
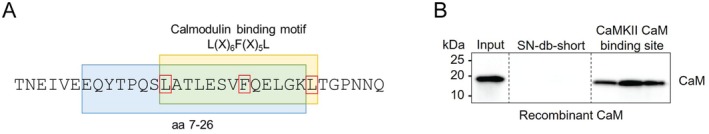
SN‐db‐short does not bind to calmodulin. (A) Schematic illustration of the calmodulin binding region in SN. The yellow box represents the calmodulin binding motif in SN. SN‐db‐short is shown in blue (amino acids 7–26). The red squares highlight the hydrophobic amino acids in the calmodulin binding motif. (B) Immunoblot analyses of CaM binding of SN‐db‐short. A peptide representing the CaM‐binding site in CaMKII was used as positive control in the pull down analysis.

We have previously shown that SN also binds to calmodulin (CaM) [11]. Sequestrating CaM away from CaMKIIδ could be an additional indirect means to inhibit CaMKIIδ. However, closer inspection of the SN‐db‐short sequence revealed that it lacks Leu27 in the calmodulin binding motif L(X)_6_F(X)_5_L (Figure 3A). To test whether SN‐db‐short still exhibited some CaM binding capacity, we performed a pull‐down experiment using SN‐db‐short and the CaM‐binding site in CaMKII (positive control) synthesised on cellulose membranes. Whereas recombinant CaM was pulled down with the positive control, no CaM was detected for SN‐db‐short (Figure 3B). Taken together, these data indicate that SN‐db‐short is a selective CaMKII inhibitor.

### 3.4 SN‐db‐Short Is Able to Internalise Into Adult Rat Cardiomyocytes

**FIGURE 4:**
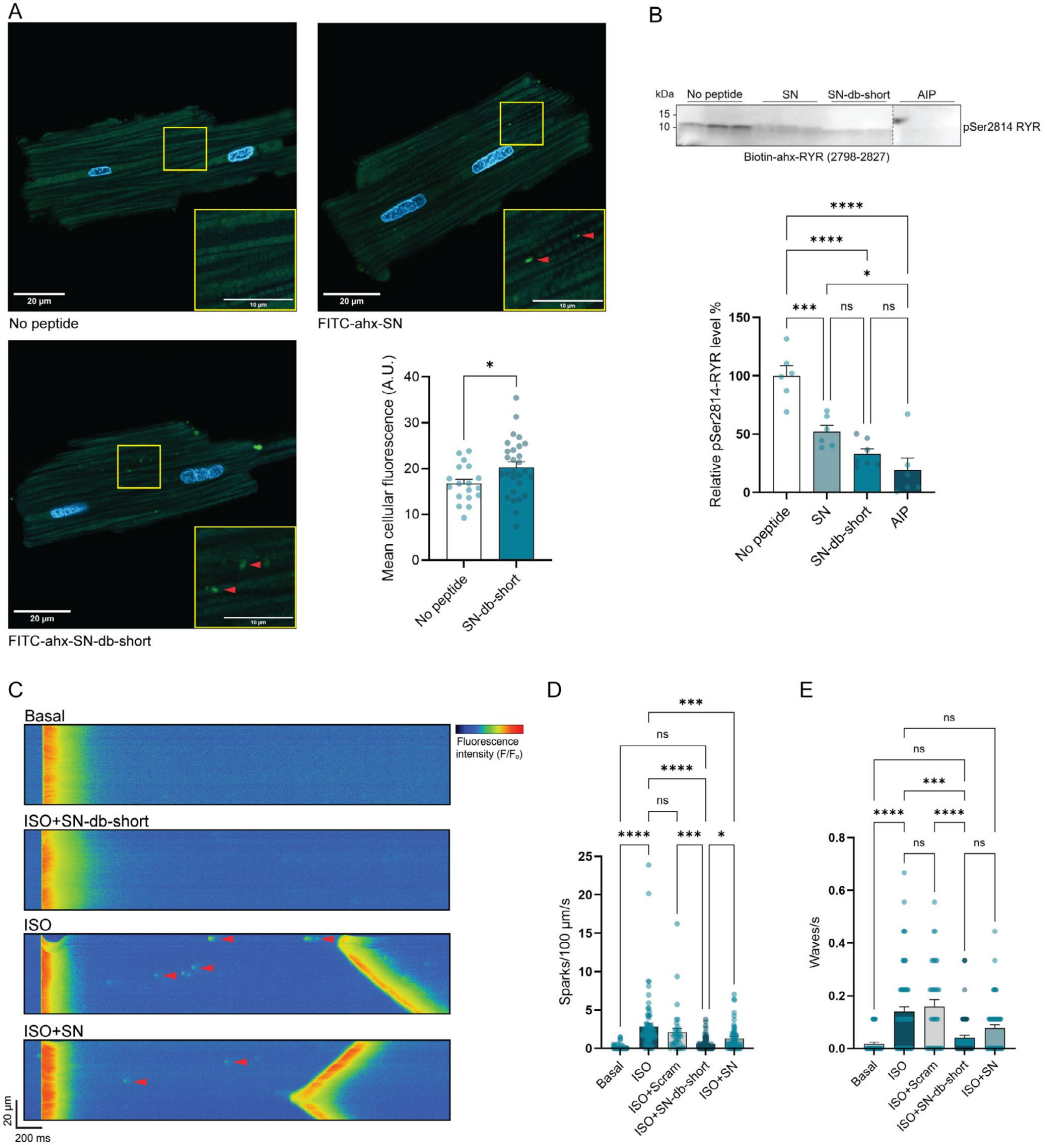
FITC‐ahx‐SN‐db‐short internalises into adult rat cardiomyocytes and inhibits CaMKIIδ‐mediated phosphorylation of Ser2814‐RYR, Ca^2+^ spark frequency and wave incidence. (A) Representative immunofluorescence images of isolated cardiomyocytes incubated with FITC‐labelled SN or FITC‐labelled SN‐db‐short for 2 h (green dots). FITC‐ahx‐SN was used as positive control and not‐treated cardiomyocytes (no peptide) as negative control. The yellow square indicates the region magnified in the bottom‐right panel. Red arrows point to the green spots. Nuclei = blue. Graph panel: Quantification of FITC fluorescence in untreated cells (No peptide) and in cells treated with SN‐db‐short. Normality of distribution was confirmed by D'Agostino and Pearson test. Significant differences were examined by unpaired *t*‐test (*n* = 18–28). *p < 0.05. (B) CaMKIIδ phosphorylation of biotin‐ahx‐RYR (2798–2827) in the presence of biotin‐SN, biotin‐SN‐db‐short and AIP (positive control). Values are presented relative to no peptide. Normality of distribution was confirmed by D'Agostino and Pearson test. Significant differences were examined by ordinary one‐way ANOVA with Tukey's multiple comparisons test (*n* = 6–7). (C) Representative Ca^2+^ sparks (red arrows) and waves of untreated cardiomyocytes (basal), and cardiomyocytes treated with ISO, ISO + SN‐db‐short and ISO + SN. (D) Ca^2+^ sparks of rat cardiomyocytes treated with or without ISO, Scram, SN‐db‐short or SN. Significant differences were examined by Kruskal–Wallis test with Dunn's multiple comparisons test (*n* = 35–85 cells from 3 to 6 rats). (E) Ca^2+^ waves of rat cardiomyocytes treated with or without ISO, Scram, SN‐db‐short or SN. Significant differences were examined by Kruskal–Wallis test with Dunn's multiple comparisons test (*n* = 35–66 cells from 3 to 5 rats). Bar charts present mean values + SEM (in B–D). *p < 0.05, ***p < 0.001, ****p < 0.0001. ns, not significant (in B, D, E).

Native SN has previously been shown to be able to internalise into cardiomyocytes [11]. To study whether the shorter and double mutated SN‐db‐short peptide still exhibited internalisation capacity, FITC‐conjugated SN‐db‐short was incubated with adult rat cardiomyocytes at a concentration of 15 μM for 2 h. Confocal microscopy analysis confirmed that FITC‐labelled SN‐db‐short internalised into rat cardiomyocytes to a similar extent as FITC‐labelled SN, with the internalised peptide visible as localised bright spots that were absent in untreated cells (Figure 4A).

### 3.5 SN‐db‐Short Inhibits CaMKII‐Mediated Phosphorylation of Ser2814‐RYR and Reduces Ca^2+^ Spark and Wave Frequency

We further investigated the inhibitory effect of SN‐db‐short on CaMKIIδ‐mediated phosphorylation of Ser2814‐RYR, using a biotinylated RYR (2798–2827) peptide as substrate. Immunoblotting with a phospho‐specific antibody showed that SN‐db‐short reduced the level of pSer2814‐RYR (Figure 4B, 67% reduction compared to no peptide, 37% reduction compared to SN). The CaMKII inhibitor AIP was used as a positive control [22].

To investigate the functional consequences of reduced Ser2814 phosphorylation, we examined the frequency of Ca^2+^ sparks and waves in adult rat cardiomyocytes. Cells were stimulated with ISO (to mimic a β‐adrenergic challenge), after pre‐treatment with SN‐db‐short, SN, or the scrambled control peptide (scram). As expected, cells treated with only ISO exhibited a higher frequency of Ca^2+^ sparks and waves compared to the basal condition, which was unaltered by the presence of the scrambled control peptide (Figure 4C–E). However, cardiomyocytes pre‐treated with SN‐db‐short showed a strong reduction in both Ca^2+^ spark and wave incidence, which were both more marked than the effect of SN treatment (Figure 4D,E).

### 3.6 SN‐db‐Short Inhibits Phosphorylation of Thr17‐PLN, and Slows Ca^2+^ Reuptake

**FIGURE 5:**
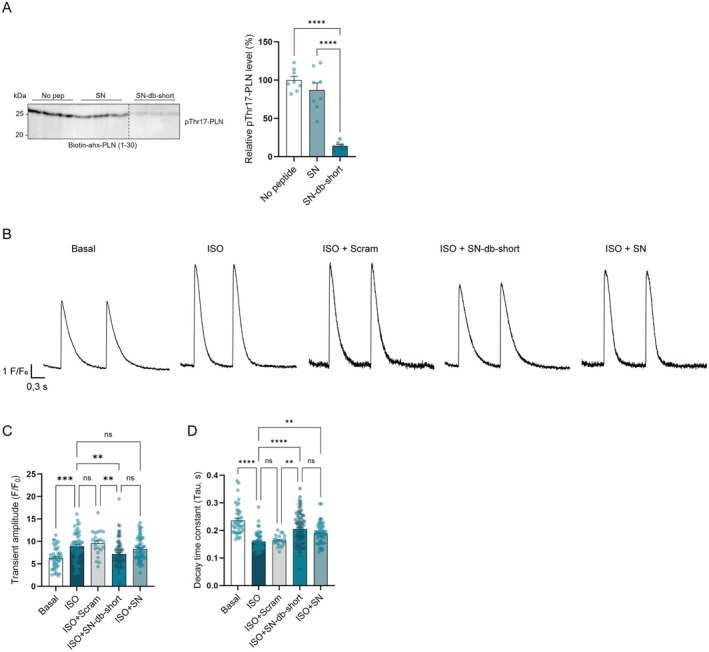
SN‐db‐short inhibits phosphorylation of Thr17‐PLN and slows Ca^2+^ reuptake. (A) CaMKIIδ‐mediated phosphorylation of biotin‐ahx‐PLN (1–30) in the presence of SN and SN‐db‐short. Values are presented relative to the negative control (no peptide). Bar charts present mean values + SEM. Normality of distribution was confirmed by Kolmogorov–Smirnov test. Significant differences were examined by ordinary one‐way ANOVA with Tukey's multiple comparisons test (*n* = 8). ****p < 0.0001. (B) Representative transients of untreated cardiomyocytes (basal), and cardiomyocytes treated with ISO, ISO + Scram, ISO + SN‐db‐short and ISO + SN. (C) Ca^2+^ transient amplitude and (D) Ca^2+^ transient decay of rat cardiomyocytes treated with or without ISO, Scram, SN‐db‐short or SN. Bar charts present mean values + SEM. Significant differences were examined by Kruskal–Wallis test with Dunn's multiple comparisons test (*n* = 22–73, 3–6 rats in B, and *n* = 22–73, 3–6 rats in C). **p < 0.01, ***p < 0.001, ****p < 0.0001, ns, not significant.

Finally, since we observed that SN can also inhibit phosphorylation of Thr17‐PLN (Figure 1A), we compared these effects with SN‐db‐short. Using biotinylated PLN (1–30), we observed that SN‐db‐short inhibited pThr17‐PLN to a far greater extent than SN (Figure 5A, 86% reduction compared to no peptide, 84% reduction compared to SN). Furthermore, experiments in field‐stimulated cardiomyocytes linked this action to alterations in Ca^2+^ transients (Figure 5B–D). Here, ISO treatment increased Ca^2+^ transient amplitude and accelerated Ca^2+^ transient decay, consistent with an expected augmentation of SERCA activity following its disinhibition by phosphorylated PLN [23]. However, these effects were significantly attenuated by SN‐db‐short, while SN showed a less pronounced effect (Figure 5B–D). Caffeine experiments revealed no change in the rate of Ca^2+^ extrusion from the cell during treatment with SN‐db‐short (Figure S3), supporting that the peptide's induced slowing of Ca^2+^ decline during the Ca^2+^ transient rather results from attenuation of SERCA activity. Taken together, these observations indicate that SN‐db‐short can attenuate CaMKII‐mediated effects on both Ca^2+^ uptake and release, and to a greater extent than SN.

## 4 Discussion

In this study, we have identified an SN derivative that inhibits and binds to CaMKIIδ more strongly than native SN. The peptide was derived from the central region of SN (amino acids 7–26), which is the most conserved domain of SN across species. Surprisingly, the shorter peptide appeared to bind CaMKIIδ more efficiently than native SN, suggesting that the N‐ and/or C‐terminus of native SN induces steric hindrance. Although the C‐terminal part of SN (aa 14–33) inhibited CaMKII to the same extent, aa 7–26 was selected for further optimisation since it demonstrated stronger CaMKII binding. Even greater affinity of this peptide for CaMKII was attained by introducing two phenylalanine mutations previously reported to increase CaMKIIδ binding to SN [10]. The final, optimised SN (7–26, L17F, V20F) peptide was named SN‐db‐short. As previously reported for native SN [10], SN‐db‐short bound to the substrate binding site (S‐site), but additional detailed mapping analysis also identified binding to the ATP‐binding region of CaMKIIδ. Modelling indicated that the two phenylalanines (Phe17 and Phe20) in SN‐db‐short formed specific favourable aromatic stacking with Phe174 in the CaMKIIδ catalytic domain.

Functionally, SN‐db short was observed to be taken up by cardiomyocytes, and it reduced CaMKIIδ‐dependent phosphorylation of Ser2814‐RYR and Thr17‐PLN. Despite this marked inhibitory action, we observed that the intracellular distribution of the fluorescently‐labelled peptide was rather sparse and punctate, suggesting that the fluorescent tag may be rapidly cleaved. Nevertheless, SN‐db‐short treatment and subsequent inhibition of CaMKIIδ phosphorylation was linked to inhibition of Ca^2+^ sparks and waves, and slowing of Ca^2+^ reuptake in adult cardiomyocytes stimulated with ISO. The role of PLN phosphorylation is relevant to the observed effects on calcium handling since during β‐adrenergic stimulation, it is phosphorylated at both serine 16 (Ser16) by PKA (protein kinase A) and at Thr17 by CaMKII. Although most believe that both sites contribute to positive lusitropy, it has been suggested that Thr17 phosphorylation may play a somewhat lesser, modulatory role, which becomes apparent under conditions of strong stimulation [24, 25]. Notably, earlier experiments using the native SN peptide showed that it selectively reduced CaMKII‐dependent Thr17 phosphorylation without affecting Ser16 phosphorylation [10, 11]. This supports the idea that SN—and very likely its optimised version, SN‐db‐short—modulates calcium handling by inhibiting CaMKII‐mediated effects, especially under high isoproterenol conditions where CaMKII activation and Thr17 phosphorylation are more prominent. Further substantiation of this point was provided by caffeine experiments, which showed a lack of effect of SN and SN‐db‐short on the activity of the Na^+^‐Ca^2+^ exchanger and plasma membrane Ca^2+^ ATPase, as indicated by the rate constant of Ca^2+^ extrusion (Figure S3). As the activity of these exchangers is also not altered by ISO‐induced phosphorylation, the data support that SN, and especially SN‐db‐short, slows Ca^2+^ transient decay in ISO‐treated cells (Figure 5D) by inhibiting PLN phosphorylation at Thr17.

CaMKII is a well‐described target in cardiovascular disease [4, 5, 6], and inhibition of CaMKII as a therapeutic strategy has been investigated in several studies [5, 7, 8, 9]. These previously described CaMKII inhibitors include small molecules such as KN‐93 [26] and ATP‐competitors [9]. Other CaMKII inhibitors comprise peptides derived from the regulatory domain of CaMKII such as AIP [22] and AC3 [27], or from the natural CaMKII inhibitor protein, CaMKIIN, such as CN21 [20]. However, in general, these CaMKII inhibitors exhibit low specificity, bioavailability, and cell permeability, and exhibit several off‐target effects. Particularly, the small KN molecules, including the well‐known KN‐93, are so‐called allosteric or ATP‐not competitive [9, 26], meaning that they are able to block CaMKII activation but not CaMKII that is autonomously active. This action is consistent with quite recent findings suggesting that KN‐93 binds to Ca^2+^/CaM, and not directly to CaMKII [28]. Not surprisingly, KN‐93 has been linked to off‐target effects on angiogenesis, cardiac remodelling, and heart failure [29]. Moreover, KN‐93 has been shown to directly inhibit the rapid component of the delayed rectifier potassium current (I_Kr_) at concentrations lower than those required for CaMKII inhibition. This additional effect on I_Kr_ may be a limitation in arrhythmia research, where KN‐93 is investigated as a potential therapeutic agent, given the critical role of I_Kr_ in the repolarisation phase of the cardiac action potential [30, 31].

Recently, several new ATP‐competitive kinase inhibitors have been reported [6, 32]. One of these, RA608, is able to overcome the issue of limited permeability and bioavailability. However, as an ATP‐competitive inhibitor, RA608 has been shown to generate off‐target effects due to the high level of conservation of the ATP binding pocket in several enzymes [32]. Another ATP‐competitive CaMKII inhibitor is the small molecule Hesperadin, which is also known for its tumour‐suppressive effects [33]. Hesperadin has demonstrated selective inhibition of CaMKIIδ compared to other CaMKII isoforms. However, its activity against additional kinases implicated in tumour progression may limit its suitability as a cardiac‐specific therapeutic. In a recent drug repurposing screen study, the small molecule Ruxolitinib was also identified as a promising ATP‐competitive CaMKII inhibitor [34]. This orally bioavailable compound is approved by the U.S. Food and Drug Administration for the treatment of myelofibrosis, polycythemia vera, and steroid‐refractory graft‐versus‐host disease. Ruxolitinib exhibits minimal off‐target effects, partially due to its limited blood–brain barrier penetration. However, since its primary role is to inhibit Janus kinases (JAK1/2), it might not be specific to CaMKII.

A number of peptide inhibitors of CaMKII have also been previously investigated, with varying levels of success. Of these, the AIP and AC3 peptides are substrate‐based inhibitors that act as substrate mimetics. However, in addition to CaMKII inhibition, they also inhibit PKD kinases [35]. Another class of inhibitors consists of peptides derived from the endogenous CaMKII inhibitor protein, CaMKIIN [36, 37] (CN21 and CN19). CN21 consists of 21 amino acids [20] and has been shown to contain the full inhibitory potency and specificity of CaMKIIN. However, the smaller CN19, which corresponds to the optimised minimal inhibitory region of CaMKIIN, exhibits even greater potency and selectivity for CaMKII [38]. On the other hand, although CaMKIIN‐derived peptides have a strong CaMKII inhibitory profile, they also interfere with other CaMKII interactors [39, 40, 41]. Recently, two peptide sequences derived from AKAP18δ were shown to inhibit CaMKII‐mediated phosphorylation of Ser2814‐RYR and thus RYR activity [19]. However, the main issues for all these peptides are low cell and tissue permeability and insufficient bioavailability [6, 32].

Our present data show that SN‐db‐short is superior to other CaMKII inhibitors as it inhibits the kinase directly through binding to the CaMKII catalytic region. This binding mode also secures inhibition of CaMKII in its auto‐phosphorylated form. Unlike other inhibitors, SN‐db‐short also internalises directly into cardiomyocytes. This action is somewhat surprising as it occurs despite the peptide's N‐ and C‐terminal truncations and the two Phe17/20 mutations. Although the internalisation route of SN‐db‐short was not investigated in detail in this study, it likely involves endocytosis as previously suggested for endogenous SN [11].

We believe that SN‐db‐short holds significant therapeutic potential for heart failure patients exhibiting sustained CaMKII activity, which places them at heightened risk for arrhythmias. This risk stems, at least in part, from an increased likelihood of spontaneous SR Ca^2+^ release events [42]. Spontaneous Ca^2+^ release from the SR contributes to the generation of delayed afterdepolarizations (DADs) through subsequent overactivation of the Na^+^/Ca^2+^ exchanger [43]. Our data show that such spontaneous SR Ca^2+^ release events are abrogated by SN‐db‐short and linked to its inhibitory roles at both the RyR and SERCA (via PLN). However, phosphorylation of L‐type calcium channels (LTCC) by CaMKII is also linked to pro‐arrhythmic early afterdepolarizations (EADs) [43]. Although we have not currently investigated whether SN‐db‐short protects against this mechanism, it seems likely based on our previous observations that full‐length SN inhibits L‐type Ca^2+^ current, EADs, and in vivo arrhythmia [10]. Based on our results to date, it is worth considering how peptides such as SN‐db‐short might be applied in patients. Recent studies have highlighted the promising use of nanoparticles as a delivery method for peptide therapeutics, particularly through inhalation, which targets cardiac tissue effectively [44, 45]. Indeed, in a porcine model of heart failure, daily inhalation of a nanoparticle‐encapsulated peptide at a low dose (0.015 mg/kg/day) over 13 days led to a ~17% improvement in left ventricular ejection fraction [45]. This therapeutic strategy for SN‐db‐short delivery will be assessed in future studies, which will also evaluate the in vivo effectiveness and pharmacokinetics of this new CaMKII inhibitor peptide.

## Author Contributions

**Ilde Rugolo:** formal analysis (lead), investigation (lead), validation (equal), visualization (lead), writing – original draft (lead), writing – review and editing (equal). **Xin Shen:** formal analysis (supporting), investigation (supporting), writing – review and editing (equal). **Thea Parsberg Støle:** formal analysis (supporting), investigation (supporting), writing – review and editing (equal). **Anna Bergan‐Dahl:** investigation (supporting), resources (equal), writing – review and editing (equal). **Ornella Manfra:** investigation (supporting), writing – review and editing (equal). **Marianne Lunde:** investigation (supporting), writing – review and editing (equal). **Geir Christensen:** conceptualization (equal), writing – review and editing (equal). **Bjørn Dalhus:** investigation (supporting), visualization (supporting), writing – review and editing (equal). **William E. Louch:** conceptualization (equal), project administration (supporting), resources (equal), writing – review and editing (equal). **Anett H. Ottesen:** conceptualization (equal), funding acquisition (equal), project administration (supporting), writing – review and editing (equal). **Helge Røsjø:** conceptualization (equal), funding acquisition (equal), project administration (supporting), supervision (supporting), writing – review and editing (equal). **Cathrine Rein Carlson:** conceptualization (equal), project administration (lead), supervision (lead), validation (equal), writing – review and editing (equal).

## Ethics Statement

All animal experiments were reviewed and approved by the Norwegian Food Safety Authority.

## Conflicts of Interest

I.R., G.C., B.D., W.E.L., A.H.O., H.R., and C.R.C. have rights in a patent application submitted by the University of Oslo related to the use of SN derivatives as a therapeutic strategy in CVD. G.C. and H.R. have intellectual property rights for the use of SN as a biomarker in CVD. The other authors declare no personal conflicts of interest.

## Supporting information


**Figure S1:** Illustration of CaMKIIδ activation. (A) The CaMKIIδ monomer contains an N‐terminal ATP binding region in its catalytic domain (in green), a regulatory domain (in pink) and a C‐terminal association (ASSN, in blue) domain allowing its oligomerisation (not shown for simplicity). In its inactive state, the threonine 287 (T287)‐segment in the regulatory domain binds to a region called the T‐site in the catalytic domain, keeping CaMKIIδ in a closed configuration. (B) Upon activation, calcified calmodulin (CaM) binds to the regulatory domain of CaMKIIδ, and displaces its interaction from the T‐site, leading to an open active conformation, allowing the kinase to phosphorylate its substrate. (C) Autophosphorylation of Thr287‐CaMKIIδ by a neighbouring monomer, keeps the kinase in an open and autonomous active configuration even after when the Ca^2+^ level is reduced, and CaM dissociates. Figure created by BioRender.com.

**Figure S2:** Confirmation of SN‐db‐short binding to human CaMKIIδ‐T287D and alignment of rat and human CaMKIIδ. (A) Left panel: Immunoblot analyses of pull‐down of human His‐CaMKIIδ‐T287D in the presence of biotin‐SN and biotin‐SN‐db‐short. Right panel: Values are presented relative to biotin‐SN. Bar charts present mean values + SEM. Normality of distribution was confirmed by D'Agostino and Pearson test. Significant differences were examined by unpaired *t*‐test (*n* = 8). ***p < 0.001. (B) Binding of biotin‐ahx‐SN‐db‐short to human His‐CaMKIIδ‐T287D. Values are presented relative to the scrambled control peptide (biotin‐ahx‐Scram). Bar charts present mean values + SEM. Normality of distribution was confirmed by Kolmogorov–Smirnov test. Significant differences were examined by an unpaired *t*‐test (*n* = 6). ***p < 0.001. (C) Alignment of rat and human CaMKIIδ. The ATP binding region, Glu97 and Glu140 in the S‐site and Ile206 and Trp238 in the T‐site in the catalytic region are shown in green, while the CaM binding region is shown in yellow. Conserved amino acids are shown in black.

**Figure S3:** Effect of SN‐db‐short on caffeine‐elicited Ca^2+^ transients. (A) Representative caffeine‐elicited Ca^2+^ transients of untreated cardiomyocytes (basal), and cardiomyocytes treated with scrambled peptide (Scram), SN‐db‐short and SN. (B) Caffeine transient amplitude (F/F_0_), (C) rate constant of Ca^2+^ extrusion (s^−1^), calculated from fits of the caffeine transient decline, in rat cardiomyocytes treated with or without Scram, SN‐db‐short and SN. Normality of distribution was confirmed by Kolmogorov–Smirnov test. Differences in caffeine transient amplitude were examined by one‐way ANOVA with Tukey's multiple comparisons test. For the rate constant of Ca^2+^ extrusion, significant differences were examined by Kruskal–Wallis test with Dunn's multiple comparisons test. (*n* = 10–18, 4–6 rats). ns, not significant.

## Data Availability

The data that support the findings of this study are available from the corresponding author upon reasonable request.
