# Supplementary figures and images for: Identification of a Small Secretoneurin Derivative That Inhibits CaMKIIδ Activity
Source: J Cell Mol Med. 2025 Oct 22;29(20):e70900. doi: 10.1111/jcmm.70900 (PMC12544700; doi:10.1111/jcmm.70900)

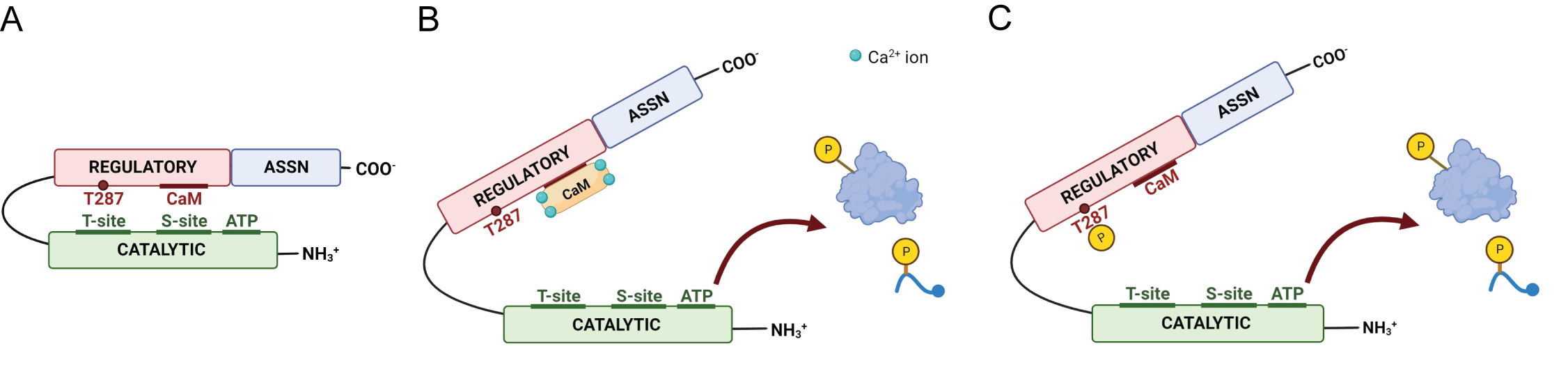

Supplement: Supplementary file 1 — Figure S1: Illustration of CaMKIIδ activation. (A) The CaMKIIδ monomer contains an N‐terminal ATP binding region in its catalytic domain (in green), a regulatory domain (in pink) and a C‐terminal association (ASSN, in blue) domain allowing its oligomerisation (not shown for simplicity). In its inactive state, the threonine 287 (T287)‐segment in the regulatory domain binds to a region called the T‐site in the catalytic domain, keeping CaMKIIδ in a closed configuration. (B) Upon activation, calcified calmodulin (CaM) binds to the regulatory domain of CaMKIIδ, and displaces its interaction from the T‐site, leading to an open active conformation, allowing the kinase to phosphorylate its substrate. (C) Autophosphorylation of Thr287‐CaMKIIδ by a neighbouring monomer, keeps the kinase in an open and autonomous active configuration even after when the Ca2+ level is reduced, and CaM dissociates. Figure created by BioRender.com. [file JCMM-29-e70900-s003.tif]

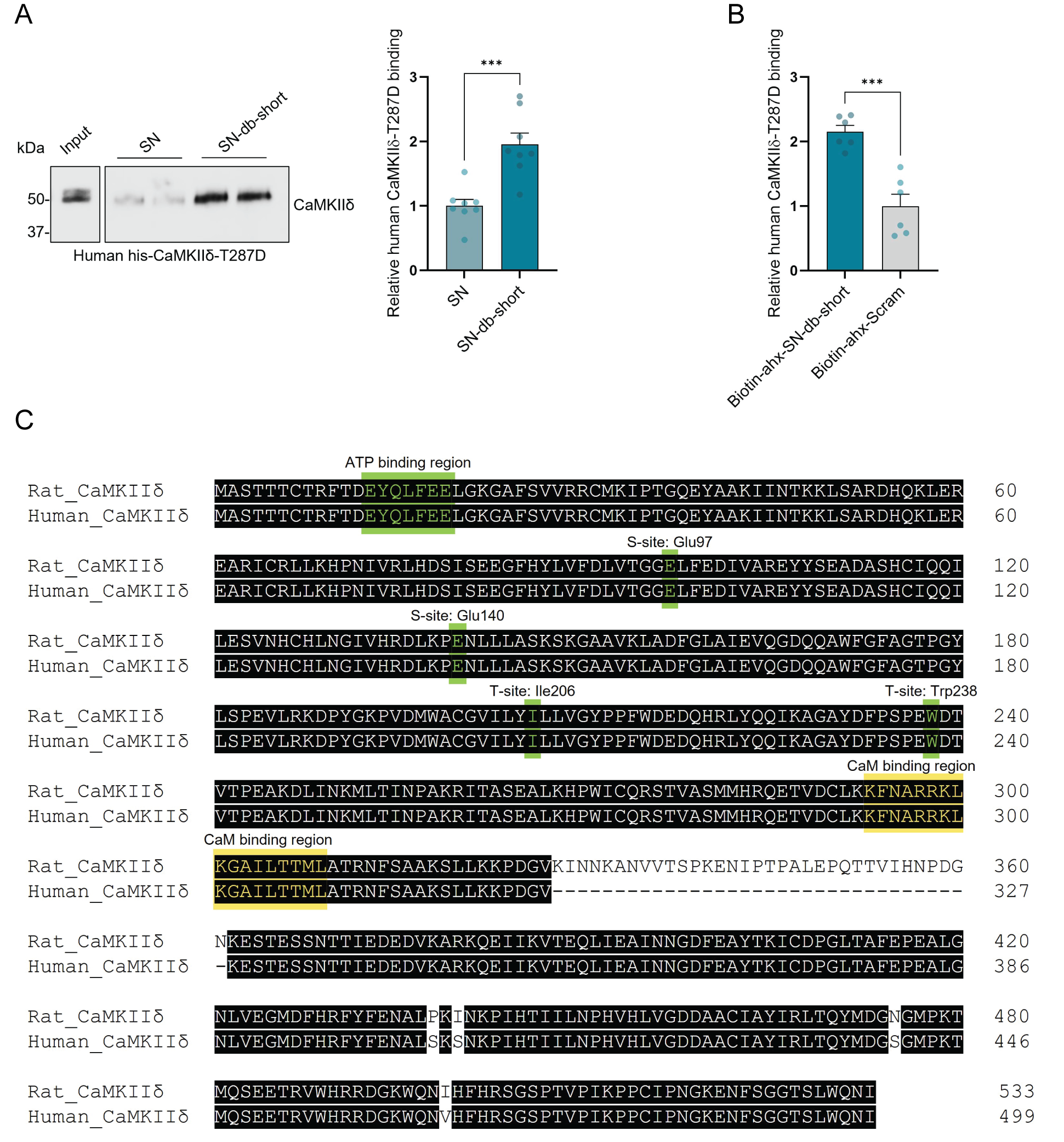

Supplement: Supplementary file 2 — Figure S2: Confirmation of SN‐db‐short binding to human CaMKIIδ‐T287D and alignment of rat and human CaMKIIδ. (A) Left panel: Immunoblot analyses of pull‐down of human His‐CaMKIIδ‐T287D in the presence of biotin‐SN and biotin‐SN‐db‐short. Right panel: Values are presented relative to biotin‐SN. Bar charts present mean values + SEM. Normality of distribution was confirmed by D'Agostino and Pearson test. Significant differences were examined by unpaired t‐test (n = 8). ***p < 0.001. (B) Binding of biotin‐ahx‐SN‐db‐short to human His‐CaMKIIδ‐T287D. Values are presented relative to the scrambled control peptide (biotin‐ahx‐Scram). Bar charts present mean values + SEM. Normality of distribution was confirmed by Kolmogorov–Smirnov test. Significant differences were examined by an unpaired t‐test (n = 6). ***p < 0.001. (C) Alignment of rat and human CaMKIIδ. The ATP binding region, Glu97 and Glu140 in the S‐site and Ile206 and Trp238 in the T‐site in the catalytic region are shown in green, while the CaM binding region is shown in yellow. Conserved amino acids are shown in black. [file JCMM-29-e70900-s002.tif]

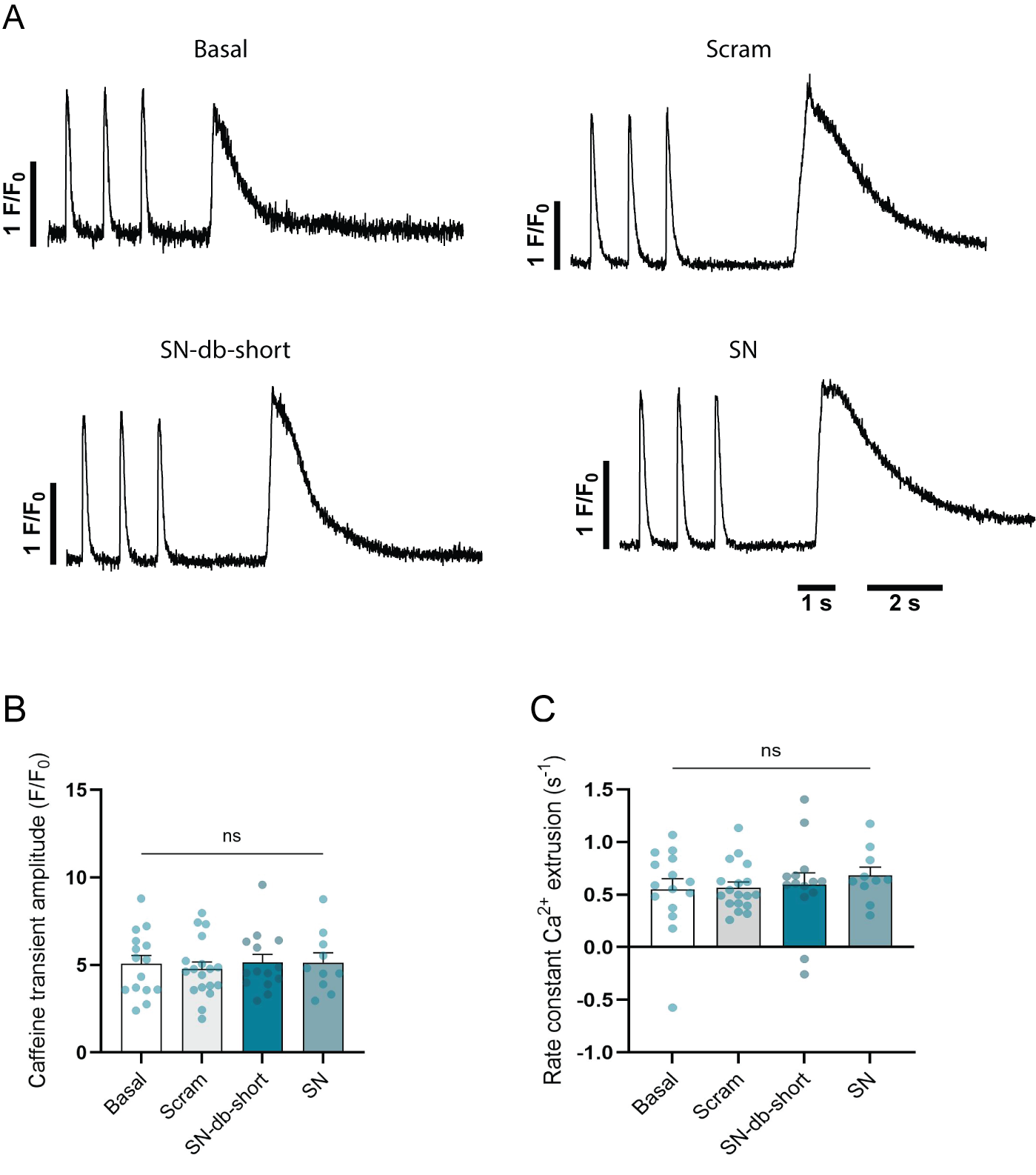

Supplement: Supplementary file 3 — Figure S3: Effect of SN‐db‐short on caffeine‐elicited Ca2+ transients. (A) Representative caffeine‐elicited Ca2+ transients of untreated cardiomyocytes (basal), and cardiomyocytes treated with scrambled peptide (Scram), SN‐db‐short and SN. (B) Caffeine transient amplitude (F/F0), (C) rate constant of Ca2+ extrusion (s−1), calculated from fits of the caffeine transient decline, in rat cardiomyocytes treated with or without Scram, SN‐db‐short and SN. Normality of distribution was confirmed by Kolmogorov–Smirnov test. Differences in caffeine transient amplitude were examined by one‐way ANOVA with Tukey's multiple comparisons test. For the rate constant of Ca2+ extrusion, significant differences were examined by Kruskal–Wallis test with Dunn's multiple comparisons test. (n = 10–18, 4–6 rats). ns, not significant. [file JCMM-29-e70900-s001.tif]
